# Prevalence of Common Mental Disorders and Associated Factors among People with Glaucoma Attending Outpatient Clinic at Menelik II Referral Hospital, Addis Ababa, Ethiopia

**DOI:** 10.1371/journal.pone.0161442

**Published:** 2016-09-01

**Authors:** Kufa Bedasso, Asres Bedaso, Fetuma Feyera, Abebaw Gebeyehu, Zegeye Yohannis

**Affiliations:** 1 Department of Psychiatry, Menelik II Health science college, Addis Ababa, Ethiopia; 2 School of Nursing and Midwifery, College of Medicine and Health Sciences, Hawassa University, Hawassa, Ethiopia; 3 Department of Nursing, College of Medicine and Health Sciences, Debre Markos University, P.O.Box: 296, Debre Markos, Ethiopia; 4 Institute of Public health, College of Medicine and health sciences, University of Gondar, Gondar, Ethiopia; 5 Department of Psychiatry, Amanuel Mental Specialized Hospital, P.O. Box: 1976, Addis Ababa, Ethiopia; Bascom Palmer Eye Institute, UNITED STATES

## Abstract

**Background:**

The burden of blindness from glaucoma is high. Therefore, people suffering from a serious eye disease such as glaucoma, which can lead to blindness, usually have an emotional disturbance on the patient. Untreated psychiatric illness is associated with increased morbidity and increased costs of care.

**Objective:**

This study aimed to assess prevalence of common mental disorders and associated factors among people with Glaucoma attending Menelik II referral hospital, Addis Ababa, Ethiopia, 2014.

**Methods:**

Institution based Cross-sectional study design was conducted in the Department of Ophthalmology Menelik II Referral Hospital from April 10 to May 15, 2014. 423 participants who had undergone through investigation, examination and diagnosed as patients of glaucoma were selected randomly from the glaucoma clinic. Data were collected through face to face interview using Self Reporting Questionnaire consisted of 20 items. Study subjects who scored ≥11 from SRQ-20 were considered as having common mental disorders. Bivariate and multivariable logistic regression analysis with 95% CI were done and variables with P<0.05 in the final model were identified as independent factors associated with common mental disorders.

**Results:**

Four hundred five patients with glaucoma were included in our study with response rate of 95.7% and 64.5% were males. The average age was 59±13.37 years. Common mental disorders were observed in 23.2% of Glaucoma patients. It is quite obvious that levels of CMDs were high among patients with glaucoma. There was a significant association between age, sex, chronic physical illness, income and duration of illness at P < 0.05.

**Conclusion and Recommendation:**

Symptoms of common mental disorders were the commonest comorbidities among patients with glaucoma. It will be better to assess and treat Common mental disorders as a separate illness in patients with glaucoma.

## Background

Common mental disorders are a group of distress states manifesting with anxiety, depressive and unexplained somatic symptoms typically encountered in community and primary care setting and also frequently occur together with a shifting combination of symptoms over time indicating emotional or mental abnormality [1]. They have been recognized as the most public health important globally, with over 450 million people worldwide estimated suffering from the problem [2].

Recently, common mental disorders account for 14% of the total burden of disease. It is predicted to be the first leading cause of disease burden by the year 2030 [3]. They have been identified as a common co-morbidities with other forms of illnesses [4–6]. Depression and anxiety constitute greater percentage of these common co-morbid psychiatric disorders. One of the illnesses, in which people suffer from depression and anxiety, is glaucoma as it can lead to blindness. It is the third cause of blindness worldwide after cataract and trachoma that has been known to leave the victims with symptoms of depression and anxiety secondary to blindness. WHO estimates about 105 million people suffer from glaucoma around the world and an estimated 5.2 million are blind from it [2]. It has been postulated that common mental disorders may impact glaucomatous disease burden and outcomes of treatment [7].

A large majority of patients with glaucoma suffer from hidden psychiatric disorders that are often undetected by their attending physicians [7]. Despite a high prevalence and burden of disease resulting from these conditions, only small proportions of people receive any form of modern treatment and most of untreated cases are found in low income countries [8]. Untreated psychiatric illness is associated with increased morbidity, a longer hospital stay and ultimately, increased costs of care [9]. This often leads to wasteful, costly and inefficient use of medical services and complications of the diagnoses.

In Ethiopia, similar to other developing countries mental health service face the same challenge [10]. Even though the national health policy of Ethiopia prioritize health care delivery at the community level, still it is not possible to address those who are suffering from mental health problem at the primary health care setting.

Studies done so far in different countries of the world [9,11–18] have shown symptoms of common mental disorders among glaucoma patients were common and associated with variety of factors which were rooted with socio-demographic and economic characteristics. However, little is known about symptoms of mental disorder. Therefore, this study aimed to assess the prevalence of common mental disorders and factors associated with it among patients with glaucoma.

## Methods and Materials

### Study Design and Setting

Institutional based cross-sectional study was conducted in Menelik II referral hospital from April 10 to May 15, 2014. Menelik II referral hospital was established in 1910 and found in Addis Ababa. Since its renovation as referral hospital, it has been serving as referral hospital for people with Glaucoma and other chronic eye disease in Ethiopia. It has different outpatient and inpatient departments. The outpatient clinics are divided in to Glaucoma clinic, anterior segment clinic, Retina clinic. Also general outpatient department for other eye disease (acute eye disease and cataract) and clinic for people with other medical disease (Hypertension and Diabetes) is available.

All patients with glaucoma who were attending glaucoma clinic at Menelik II referral hospital considered as source population. Those who were attend glaucoma clinic at Menelik II referral hospital during the study period and fulfilled inclusion criteria were included in the study. Clients who were unable to communicate to for the interview as a result of critical illness were excluded from this study.

### Sample Size Determination and Sampling Procedures

Sample size was determined by using single population proportion formula considering the following assumptions

$$n=\frac{\left(Z\alpha ⁄2{)}^{2}\right)P\left(1-P\right)}{{\text{d}}^{2}}$$

n = minimum sample size required for the study

Z = standard normal distribution with confidence interval of 95%, Z = 1.96

d = Absolute precision or tolerable margin of error. d = 0.05

P = is the anticipated population proportion

Since no similar study conducted previously in Ethiopia, 50% was used to anticipate the proportion of the population of glaucoma patients who experience common mental disorder.

$$\text{Therefore}\;n=\frac{0.5\left(1-0.5\right)1.96^{2}}{{\left(0.05\right)}^{2}}=384$$

Adding 10% non-response rate, the final sample size was **423**.

A systematic random sampling technique was used to select the respondents for interview. A total of 12,576 and 1048 clients attend glaucoma clinic annually and monthly respectively. The interval was obtained by dividing the average number of clients attending the clinic with a month by the sample size as 1048 ⁄ 423 which was 3. The first individual was selected using lottery method and the others were selected at a regular interval by systematic random sampling method.

### Measurements

Data were collected by using SRQ-20 whether; the respondents had experienced symptoms associated with emotional distress within 4 weeks before the interview. The tool was developed by WHO to screen common mental disorder in primary health care setting in low income countries [19]. The SRQ-20 was translated and validated for use in Ethiopia [20]. Those who answered ‘yes’ to eleven questions or more on the SRQ-20 were defined as having mental distress. Semi-structured questionnaire was used to collect socio demographic, substance use and clinical factors. Current use of substance was considered for the last three months.

Three diploma nurses who received a two days intensive training on data collection techniques were involved in data collection. Pre-test was done at Zewuditu hospital in 5% of sample and Cronbach’s alpha was determined for the tool to check the reliability of the tool. The value of Cronbach’s alpha was >0.7 for the entire tool. Supervision was held during data collection and each questionnaire was checked for completeness by supervisor on daily basis.

### Data Processing and Analysis

Collected data were entered in to Epi-Info version 7 and exported to SPSS version 20 for analysis. Descriptive statistics was used to identify distributions of socio-demographic characteristics of study participants. Prevalence estimates of mental distress were calculated separately for men and women. The distribution of SRQ-20 scores among men and women, as well as the prevalence of each specific symptom were also estimated. Both bivariate and multivariable logistic regression analysis with 95% CI were used to see the association between each independent variables and common mental disorders. Finally variables which showed statistical significance at P<0.05 and 95% CI in the final model were reported as independently associated with CMDs.

### Ethical Statement

Ethical clearance was obtained from ethical review board of University of Gondar, College of Medicine and Health Sciences, institute of Public health and Amanuel mental specialized hospital. Ethical review board approved the methods of data collection and forms of consent. After thoroughly discussing the ultimate purpose and method of the study, a written consent was sought from Addis Ababa health bureau and written informed consent was obtained from each study participant who can read and write. After reading the consent statement by the data collectors, finger prints were obtained from those participants who could not read and write. The respondents were informed that their inclusion in the study is voluntary and they are free to withdraw from the study if they are not willing to participate. If any question they do not want to answer they have the right to do so. Anonymity was considered to ensure confidentiality of respondents.

## Results

The study included 423 individuals, among these 18 (12 questionnaires found to be incomplete and 6 individuals refused to be interviewed) excluded from the analysis and registered as non-response.

### Socio-Demographic, Substance Use and Clinical Characteristics of Study Participants

405 interviews were completed with the overall response rate of 95.7%. The mean age of the clients was 59 ± (13.37) years. The majority were men (64.5%), married (69.9%), orthodox Christian (84.2%), unemployed (54.1) and from urban (83%). Half (50.1%) of the participants were Amhara and 38.5% had no formal education. Minorities (10.6%) were current substance user, 17.8% were reported having a hypertension disease and 72.1% of clients were living with glaucoma for more than 12months. (Table 1).

**Table 1:** Socio-demographic characteristics, substance use and clinical factors of study subjects among adult patients attended glaucoma clinic at Menelik II referral hospital, Addis Ababa, Ethiopia, 2014.

Characteristics	Numbers	Percent
**Sex**		
**Male**	260	64.2
**Female**	145	35.8
**Age**		
**20–39**	33	8.1
**40–59**	150	37
**>60**	222	54.8
**Marital status**		
**Married**	283	69.9
**Single**	39	9.6
**Divorced**	29	7.2
**Widowed**	54	13.3
**Educational status**		
**No formal educations**	156	38.5
**Primary school**	100	24.7
**High school**	85	21
**Higher education**	64	15.8
**Religion**		
**Orthodox**	341	84.2
**Muslims**	33	8.1
**Others**	31	7.7
**Occupational status**		
**Employed**	126	31.1
**Unemployed**	279	68.9
**Ethnicity**		
**Oromo**	129	31.9
**Amhara**	203	50.1
**Tigre**	20	4.9
**Others**	53	13.1
**Residence**		
**Urban**	336	83
**Rural**	69	17
**Income**		
**<750ETB**	305	75.3
**750-1200ETB**	31	7.7
**>1200ETB**	69	17
**Substance ever user**		
**No**	376	92.3
**Yes**	29	7.2
**Substance current user**		
**No**	362	89.4
**Yes**	43	10.6
**Chronic physical illness**		
**Hypertension only**	72	17.7
**Diabetes only**	29	7.2
**Hypertension and diabetes**	48	11.9
**No chronic illness**	256	63.2
**Duration of illness**		
**≤12 months**	113	27.9
**>12months**	292	72.1

### The Prevalence of Common Mental Disorder Symptoms

Ninety four (23.21%) study subjects scored ≥11 on SRQ-20 from the total of 20. Women had higher prevalence (33.1%) of all mental distress symptoms compared with men (17.7%). Sleeping badly (38.7%) and frequent headache (60.7%) were the most common symptoms reported among women while being easily tired (28.8%) and having frequent headache (35.4%) were more common symptoms experienced by men. (S1 Table).

### Factors Associated with Common Mental Disorders

The likelihood of CMDs was about two folds higher among women as compared with men (AOR = 1.83, 95%CI: 1.05–3.18). Subjects who were 20–39 years had 8 folds increased risk of CMDs compared to those who were 60 years and older, (AOR = 8.59, 95%CI: 3.36–21.94). The odds of having common mental disorders for subjects who were 40–59 years were two times higher than the odds of subjects who were 60 years and above, (AOR = 2.20, 95%CI: 1.18–4.07). The likelihood of common mental disorders was three and half folds higher among subjects with monthly income <750ETB as compared to those who were with income >1200ETB per month (AOR = 3.51, 95%CI: 1.53–7.99).

Participants reporting chronic physical illness (hypertension and diabetes) were more likely to have CMDs compared with clients without chronic illness. Respondents who had hypertension were about three times more likely to experience CMDs compared with those who had no chronic physical illness (AOR = 2.93, 95%CI: 1.45–5.91). The diabetic clients were about five times more likely to develop CMDs compared with client without chronic physical illness (AOR = 5.23, 95%CI: 2.09–13.39). Subjects with combined hypertensive and diabetic disorders were observed to have increased odds of CMDs (AOR = 17.14, 95%CI: 7.58, 38.75).

Durations of the illness were significantly associated with CMDs. The odds of having common mental disorders for clients less than 12months durations of illness was two times higher as compared to those greater than 12months duration of illness (AOR = 2.27,95%CI: 1.3–3.96).

On the other hand substance use, religion, marital status, level of education and occupational status didn’t show statistically significant association with common mental disorder in this study. (Table 2).

**Table 2:** Bivariate and Multivariate logistic analysis result of study subjects among adult patients attend glaucoma clinic at Menelik II referral hospital, Addis Ababa, Ethiopia, 2014.

Variables	CMDs		COR (95%CI)	AOR(95%CI)
	Yes	No		
**Sex**				
Male	46	214	1	1
vFemale	48	97	2.30(1.44–3.68)	1.83(1.05–3.18)**
**Age**				
20–39	12	21	2.45(1.12–5.37)	8.59(3.36–21.94)*
40–59	40	110	1.56(0.95,2.55)	2.20(1.18–4.07)*
>60	42	180	1	1
**Marital status**				
Married	55	228	0.63(0.32–1.23)	1.13(0.49–2.64)
Single	16	23	1.81(0.76–4.33)	2.16(0.65–7.18)
Divorced	8	21	0.99(0.36–2.72)	0.69(0.21–2.30)
Widowed	15	39	1	1
**Educational status**				
No formal education	39	117	1.44(0.70–2.98)	2.09(0.76–5.74)
Primary school	19	81	1.02(0.46–2.27)	0.95(0.34–2.59)
Secondary school	24	61	1.75(0.78–3.74)	1.32(0.51–3.41)
Higher education	12	52	1	1
**Occupational status**				
Employed	26	100	1	1
unemployed	68	211	1.24(0.74–2.07)	0.79(0.36–1.73)
**Monthly income**				
<750ETB	78	227	1.48(0.77–2.85)	3.51(1.53–7.99)*
750-1200ETB	3	28	0.46(0.12–1.75)	0.58(0.12–2.80)
>1200ETB	13	56	1	1
**Residence**				
Rural	14	55	0.82(0.43–1.54)	0.48(0.20–1.12)
Urban	80	256	1	1
**Chronic physical illness**				
Hypertension only	19	53	2.19(1.16–4.12)	2.93(1.45–5.91)*
Diabetes only	11	18	3.74(1.63–8.55)	5.23(2.09–13.39)*
Hypertension and diabetes	28	20	8.56(4.36–16.77)	17.14(7.58–38.75)*
No chronic physical illness	36	220	1	1
**Duration of illness**				
≤12 months	37	76	2.01(1.23–3.27)	2.66(1.48–4.78)*
>12 months	57	235	1	1

** P<0.01,

* P<0.05

## Discussion

This study had assessed common mental disorders using SRQ-20. From the total of 20 possible score measured by the tool, score of ≥11 was observed in 94 (23.21%) of study subjects. Almost similar finding was reported in Kenya (21.7%) [21] and Nigeria (22%) [14] and relatively higher than reported in hospital based studies among general medical disease in different departments in Ethiopia (6.8–18%) [22]. The higher prevalence in the current study might reflect the particular psycho-social stresses experienced by this group of patients.

Women were two times more likely to experience CMDs compared with men. This findings is consistent with studies conducted in Brazil and India respectively [17,18]. It has been shown that mental health problems particularly depression, anxiety and somatic complaints affect women to a greater extent than men across diverse societies and social contexts [14]. Proportionally, symptoms of CMDs were more common in women compared with men across all age groups. The reason might be pressures from their multiple roles, gender based discrimination and based violence; contribute to women's poor mental health [23].

Common mental disorders were significantly associated with age. Study subjects aged between 20–39 years were 8.57 times more likely to develop CMDs as compared with those 60 years and above. The odds ratio of CMDs was more than two-fold higher among age group 40 to 59 years as compared with those 60 years and above. The finding in this study was to the contrary, and disagrees with the findings of many other studies. The possible reason might be as it was known glaucoma is a disease which can result in bilateral blindness, as a result patients may become anxious about losing their job and becoming unable to earn their living [24].

Common mental disorders were significantly associated with chronic physical illness. Hypertensive patients were three times more likely to report CMDs as compared with those without chronic physical illness. Diabetic illness had significant association with CMDs. Similarly those who were with diabetes and hypertension illness were 17.14 times more likely to develop CMDs as compared with those without chronic illness. The reason might be those living with chronic physical illness might have limited activity, experience dissatisfaction in life which may expose them for depression and anxiety. The finding of this study is in line with study done in Brazil and India [17,18] respectively.

Low income had significant association with common mental disorders. Subjects who got below 750 ETB were 3.5 times more likely to develop common mental disorders as compared to those who got more than 1200 ETB. This might be an insufficient income lead to stressful and unsafe situation, which may trigger CMDs. People experiencing poverty face difficulties to fulfill basic needs, unoffered the treatment and it interfere with their ability to participate in productive activity. This finding is consistent with the study done in Brazil [17]. Higher levels of hopelessness, lack of control over their circumstance, an orientation towards the present rather than the future and lower level of satisfaction with life are some of the psychological impact of low socioeconomic status [25,26].

In this study an increased duration of illness from glaucoma has been found to have a less relative risk of CMDs compared with those who were with short duration of illness from glaucoma. Subjects with illness from glaucoma for the duration of 12 months or less were 2.66 times more likely to develop CMDs as compared with those who lived for more than 12 months with the illness. The level of comprehension of patients with glaucoma could have an impact on the clients to cope up with the illness. Those who lived for longer duration from glaucoma might have better coping mechanism and hence less likelihood of CMDs compared to those who lived for short duration. A report from the study done in China also revealed duration of glaucoma illness and comprehension were positively correlated [27].

### Limitations of the Study

Recall bias could have been encountered since this study used self-reported questionnaire (SRQ-20).

### Conclusion and Recommendations

Common mental disorders have been found prevalent among patients with glaucoma. This result showed common mental disorders were closely linked with socio-demographic characteristics (age, sex income) among patients with glaucoma in which young patients, females and those with low income were found to be more vulnerable. In addition presences of additional concomitant chronic physical morbidities were found to synergize the occurrence of common mental disorders among patients with glaucoma.

This study has indicated a need for the integration of psychosocial care into current medical treatment of patients with glaucoma. Better to treat Common mental disorders as a separate illness in patients with glaucoma. This could be attained if health professionals working at glaucoma and other chronic physical illness clinics are given Mental Health gap action program (mh-GAP) training.

## Supporting Information

S1 Table Symptoms of common mental disorders measured by SRQ-20 among adult patients attended glaucoma clinic at Menelik II referral hospital, Addis Ababa, Ethiopia, 2014. (DOCX)
