# Supplementary material for: Prevalence of Common Mental Disorders and Associated Factors among People with Glaucoma Attending Outpatient Clinic at Menelik II Referral Hospital, Addis Ababa, Ethiopia
Source: PLoS One. 2016 Sep 1;11(9):e0161442. doi: 10.1371/journal.pone.0161442 (PMC5008864; doi:10.1371/journal.pone.0161442)
Supplement: S1 Table — (DOCX) [file pone.0161442.s001.docx]

**S1** **Table.** Symptoms of common mental disorders measured by SRQ-20 among adult patients attended glaucoma clinic at Menelik II referral hospital, Addis Ababa, Ethiopia, 2014

| **SRQ-20 items Men (n=260) Women (n=145)** | | | | |
| --- | --- | --- | --- | --- |
|  | **Number** | **Percent** | **Number** | **Percent** |
| Frequent headache | 92 | 35.4 | 88 | 60.7 |
| Poor appetite | 48 | 18.5 | 52 | 35.9 |
| Sleeping badly | 61 | 23.5 | 56 | 38.6 |
| Easily startled | 47 | 18.1 | 46 | 31.7 |
| Hand shake | 45 | 17.3 | 39 | 26.9 |
| Nervous, tense and worries | 52 | 20 | 50 | 34.5 |
| Poor digestion | 51 | 19.6 | 51 | 35.2 |
| Having trouble thinking | 45 | 17.3 | 42 | 29 |
| Feeling sadness | 47 | 18.1 | 45 | 31 |
| Crying in unusual way | 42 | 16.2 | 33 | 22.8 |
| Daily work suffering | 21 | 8.1 | 19 | 13.1 |
| Difficult in reaching decision | 17 | 6.5 | 12 | 8.3 |
| Problem in engaging in daily activities | 21 | 8.1 | 19 | 13.1 |
| Unable to play useful part in life | 13 | 5 | 6 | 4.1 |
| lost interest in things | 18 | 6.9 | 27 | 18.6 |
| Feeling worthless | 12 | 4.6 | 15 | 10.3 |
| Having suicidal ideation | 14 | 5.4 | 13 | 9 |
| Feeling tired all the time | 45 | 17.3 | 37 | 25.5 |
| Uncomfortable feeling in stomach | 57 | 21.9 | 52 | 35.9 |
| Easily tired | 75 | 28.8 | 64 | 44.1 |
